# Kyphosis Correction in Patients Undergoing a Four-Level Anterior Cervical Discectomy and Fusion

**DOI:** 10.7759/cureus.8826

**Published:** 2020-06-25

**Authors:** James Ebot, Stephanie Foskey, Ricardo Domingo, Eric Nottmeier

**Affiliations:** 1 Neurological Surgery, Mayo Clinic, Jacksonville, USA

**Keywords:** acdf, kyphosis, lordosis, deformity

## Abstract

Introduction

Cervical kyphotic deformity can be quite debilitating. Most patients present with neck pain, but they can also present with radiculopathy, myelopathy, altered vertical gaze, swallowing problems, and even cosmetic issues from the severe kyphotic deformity. After failing conservative management, surgery remains the only option for halting symptom progression. Surgical options for cervical kyphosis have included anterior-only approaches, posterior-only approaches, or 360- and 540-degree reconstructions. This paper addresses the correction of cervical kyphotic deformity via an anterior-only approach consisting of a four-level anterior cervical discectomy and fusion (ACDF).

Methods

We interrogated our procedure log system and the keyword “anterior cervical discectomy and fusion (ACDF)” was typed into the search bar. All patients with an ACDF for the past five years were reviewed and patients with a four-level ACDF were selected. Chart review was performed and patients presenting with multi-level cervical stenosis with kyphosis were included in the study. Pre- and post-surgery images were reviewed, and the degrees of pre-operative kyphosis and post-operative lordosis were measured.

Results

Our search produced 20 patients. All the patients had a diagnosis of multi-level cervical stenosis with or without myelopathy and were all symptomatic. Pre-operative kyphosis ranged from 2.3 to 35 (mean 11.5) degrees, and post-operative lordosis ranged from 2 to 38 (mean 16) degrees. All the patients had varying degrees of kyphosis correction post-surgery which ranged from 6 to 44 (mean 27) degrees. Significant improvement or complete resolution of symptoms post-operatively occurred in all patients.

Conclusion

Four-level ACDF in carefully selected patients can be used to correct cervical alignment in patients presenting with symptomatic multi-level cervical stenosis with kyphosis.

## Introduction

Cervical kyphotic deformity can be quite debilitating. Most patients present with neck pain, but they can also present with radiculopathy, myelopathy, altered vertical gaze, swallowing problems, and even cosmetic issues from severe kyphotic deformity [1]. The presence of normal cervical lordosis can be attributed to the relatively increased disc height anteriorly compared to posteriorly. Progressive loss of this disc height anteriorly over time either due to degenerative processes or from iatrogenic causes can lead to cervical kyphosis. Other causes can include trauma, malignancy, infection, or inflammatory processes [2]. After failing conservative management, surgery remains the only option for halting symptom progression. Surgical options for cervical kyphosis have included anterior-only approaches, posterior-only approaches, or 360- and 540-degree reconstructions [2].

Anterior cervical discectomy and fusion (ACDF) is a common neurosurgical procedure used to treat cervical degenerative disc disease. It is routinely performed for one- and two-level cervical disc disease in patients presenting with cervical radicular symptoms or myelopathy. Surgical outcomes and symptom resolution are typically acceptable in patients undergoing one- and two-level ACDF [3]. For patients presenting with multi-level cervical disc disease, a three- to four-level ACDF may be necessary to address all levels involved. The anterior approach is important in patients with cervical kyphosis as it plays an important role in correcting the kyphotic deformity [4]. However, in patients undergoing a four-level ACDF, some authors recommend an added posterior approach to aid with arthrodesis [5]. The benefits of an anterior-only approach include shorter operating room (OR) times, decreased blood loss intra-operatively, less consumption of opioid medications, and shorter hospital stay [6,7]. This paper addresses the correction of cervical kyphotic deformity via an anterior-only approach consisting of a four-level ACDF.

## Materials and methods

**Figure 1:**
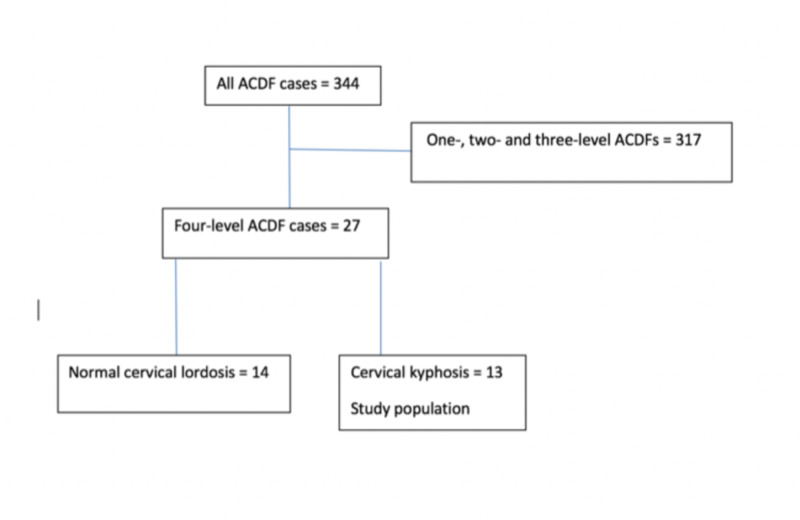
Patient selection algorithm Flowchart showing our patient selection criteria. Only patients with a pre-operative kyphosis as measured by the C2-C7 Cobb angle were included in the study. ACDF, anterior cervical discectomy and fusion.

Institutional review board approval was obtained. A retrospective chart review was performed and all patients undergoing cervical kyphosis correction via a four-level ACDF by the senior author (E.W.N.) were reviewed. Surgeries were performed in Jacksonville, FL at either Mayo Clinic Florida or the St. Vincent’s Spine and Brain Institute. We interrogated our procedural log system and the key phrase “anterior cervical discectomy and fusion” was typed into the search bar. All patients undergoing ACDF within the past five years were identified, and patients with a four-level ACDF were selected. A detailed chart review was performed, and patients presenting with multi-level cervical stenosis with kyphosis were selected for the study (Figure 1). Pre- and post-surgery images were reviewed, and the degree of pre-operative kyphosis and post-operative lordosis were measured using the C2-C7 Cobb angle. Cobb angles were measured using radiographic viewing software on plain images.

## Results

Our search initially produced 344 ACDF patients. After eliminating patients undergoing one-, two-, and three-level ACDFs, 27 patients were identified who underwent a four-level ACDF. Seven of the 27 patients did not meet the criteria for cervical kyphosis pre-operatively, defined as a negative C2-C7 Cobb angle, and were eliminated from the study. This produced a total of 20 patients (Table 1) who qualified for the study and had varying degrees of pre-operative cervical kyphosis. The average follow-up was 14 months (1-62 months). All the patients had a diagnosis of symptomatic cervical spondylosis with or without myelopathy. Pre-operative cervical spine alignment as measured by the C2-C7 Cobb angle ranged from 2.3 to 35 (mean 11.5) degrees kyphosis, and post-operative cervical spine alignment ranged from 2 to 38 (mean 16) degrees lordosis. All the patients had varying degrees of kyphosis correction which ranged from 6 to 44 degrees (mean 27). All patients had either significant improvement or complete resolution of their symptoms post-operatively. Ten patients had completed a 12-month follow-up CT scan which showed a C6-7 pseudoarthrosis in seven of the ten patients, yielding a radiographic non-union rate of 70% for this small cohort (Table 1). All seven patients with pseudoarthrosis remained asymptomatic and did not require a posterior reinforcement. There was no correlation between radiographic non-union and Cobb angle correction post-operatively. One patient reported post-operative dysphagia which resolved completely after four months. 

**Table 1:** Change in cervical alignment following a four-level anterior cervical discectomy and fusion Patients were considered fused at their 12-month follow-up imaging if they demonstrated continuous bridging bone formation at all four disc levels, with no motion on dynamic imaging.

Patients	Cervical kyphosis pre-operative (degrees)	Cervical lordosis post-operative (degrees)	Change in alignment (degrees)	Follow-up (months)	12-month follow-up CT scan
1	33	2.5	35.5	5.5	Pending
2	16	15	31	43	C6-7 pseudoarthrosis
3	35	3	38	6	Pending
4	12	7	19	62	Fused
5	5	2	7	6.5	Pending
6	15	8.5	23.5	41	C6-7 pseudoarthrosis
7	10	34	44	1	Pending
8	15	21	36	8	Pending
9	6	8	14	12	Fused
10	3	30	33	13	C6-7 pseudoarthrosis
11	8	23	31	2	Pending
12	4	38	42	5	Pending
13	22	17	39	12	C6-7 pseudoarthrosis
14	3	3	6	12	Fused
15	2.3	10	12.3	12	C6-7 pseudoarthrosis
16	8.8	31	39.8	3.5	Pending
17	6.2	4.2	10.4	16	C6-7 pseudoarthrosis
18	16	10	26	2.5	Pending
19	32.5	10.6	43.1	6	Pending
20	8	9	17	12	C6-7 pseudoarthrosis

## Discussion

**Figure 2:**
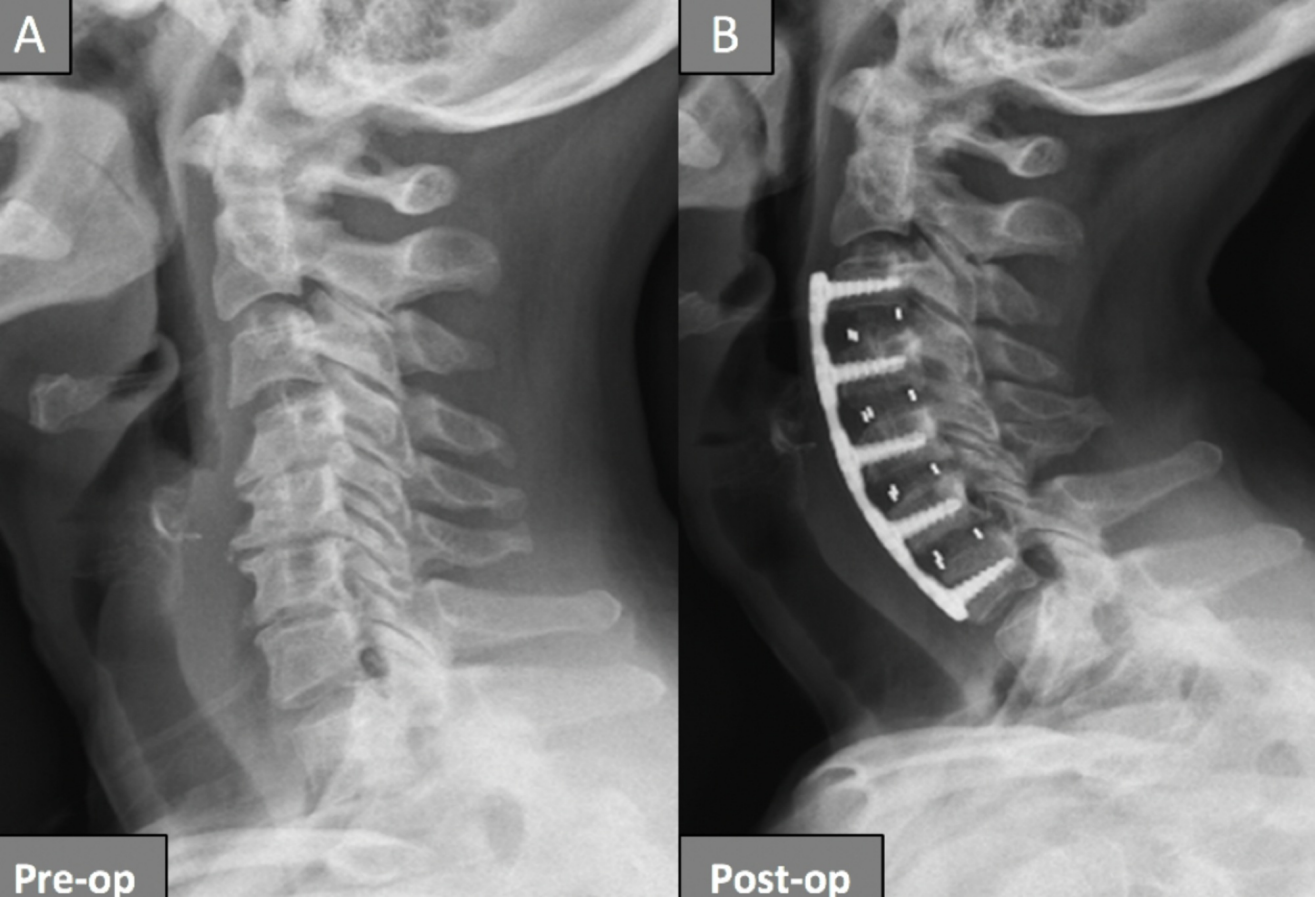
Pre- and post-operative plain films of a patient who underwent a four-level anterior cervical discectomy and fusion for kyphosis correction Plain films of a patient before surgery (A) showing loss of cervical lordosis with development of kyphosis. The same patient patient is shown after undergoing a four-level anterior cervical discectomy and fusion with kyphosis correction, showing good cervical lordosis (B). Pre-op, pre operative; Post-op, post-operative.

The management of cervical spine degenerative disc disease continues to evolve. Conservative management is the treatment of choice for patients presenting with mostly neck pain and no neurologic deficits [8]. For patients presenting with severe radiculopathy or myelopathy, or in patients failing conservative treatment, surgery is the next option. The surgical approach depends on a variety of factors, including the pathology, specific anatomy, or even the comfort level of the surgeon with the approach. Correction of cervical kyphosis typically involves a 360-degree or 540-degree cervical reconstruction [2]. Our study assessed correction of cervical kyphosis in symptomatic patients undergoing stand-alone four-level ACDF by a single surgeon. All the patients in our series had improvement or complete resolution of their preoperative symptoms at the time of this study. This validates previous studies demonstrating good patient outcomes using the anterior approach, especially when the pathology is mostly anterior. Additionally, an anterior-only approach avoids the muscle dissection used in posterior cervical approaches, thereby decreasing post-operative pain and overall length of hospital stay. Better kyphosis correction with an anterior compared to a posterior approach has been reported in the literature [6]. In our patient cohort, kyphosis correction was accomplished by a combination of the use of lordotic cages and plates. We were able to show varying degrees of kyphosis correction in this study from the anterior-only approach (Figure 2). None of the patients required a posterior revision during the review period.

A number of review studies have documented safe and good surgical outcomes with four-level ACDFs [7,9]. Some review studies have shown a wide range of post-operative dysphagia with ACDFs, with increased frequency of occurrence in patients undergoing three- and four-level ACDFs, more than 50% incidence [7,10]. Some retrospective studies have shown a low incidence of post-operative dysphagia in patients undergoing a four-level ACDF [7]. In our series, we recorded one out of the 20 patients with post-operative dysphagia which resolved completely at four months post-operatively. Some studies have shown high pseudoarthrosis with three- and four-level ACDFs compared to one- and two-level ACDFs. While most patients in this study with available follow-up CT scans showed pseudoarthrosis at C6-7, they all remained asymptomatic and did not require a posterior revision. Long-term follow-up is required in this domain and can help inform practices who advocate for a posterior reinforcement when performing a four-level ACDF.

Loss of cervical lordosis has been associated with neck pain and muscle cramps [11]. The added work placed on the muscles to maintain a normal plain of sight can lead to muscle fatigue overtime, which can manifest as neck pain. Patients presenting with degenerative cervical spine disease may have varying levels of cervical kyphosis, and it is uncertain how much added neck pain is due to their cervical malalignment. Treating their presenting pathology may lessen their pain, but some patients may continue with persistent chronic neck pain with no radiographic abnormality except for cervical malalignment. Therefore, it is important to assess the cervical spine alignment in these patients and to aim for optimal correction during surgery in patients presenting with cervical spondylosis with kyphosis.

## Conclusions

Degenerative cervical spine disease is a very common pathology seen by spine surgeons. Surgical management is variable and usually tailored to the unique patient presentation. We show in our series that for carefully selected patients with no posterior pathology to address, a multi-level ACDF can be safely used to address cervical degenerative disc disease while also correcting the cervical spine alignment.
